# A cell comparative multiple instance learning network guided by image quality assessment for cervical whole slide image classification

**DOI:** 10.1016/j.isci.2026.115561

**Published:** 2026-04-01

**Authors:** Lanlan Kang, Jian Wang, Yongjun He, Jian Qin

**Affiliations:** 1 School of Computer Science and Technology, Anhui University of Technology, Maanshan, Anhui Province, China; 2 School of Computer Science and Technology, Harbin University of Science and Technology, Harbin, Heilongjiang Province, China; 3 School of Computer Science and Technology, Harbin Institute of Technology, Harbin, Heilongjiang Province, China

**Keywords:** Bioinformatics

## Abstract

Early screening is essential for reducing the incidence and mortality of cervical cancer, and artificial intelligence-based analysis of whole slide images (WSIs) enables large-scale automated screening. However, existing methods often ignore image quality variations and inter-individual morphological differences, which limits their robustness in clinical settings. This study proposes a quality-aware cervical WSI classification framework that integrates image quality assessment with pathologist-inspired normal-abnormal cell comparison. A quality evaluation module filters unreliable patches, while a cell comparison and enhancement strategy enlarges the feature discrepancy between normal and abnormal cells to mitigate individual variability. Supervised contrastive learning further strengthens abnormal cell discrimination, and patch-level quality scores are incorporated into an attention-based multiple instance learning framework to guide WSI classification. Experiments on 2,434 WSIs from five medical institutions demonstrate that our method achieves superior performance in real-world scenarios, significantly outperforming state-of-the-art methods by 1.93% in average overall accuracy.

## Highlights

• A WSI classification method mimicking pathologists by fusing cell comparison and IQA
• First to introduce IQA into cervical WSI classification, used as MIL weights
• Validated on 2,434 multi-center WSIs, achieving pathologist-level performance

## Graphical abstract

**Figure:**
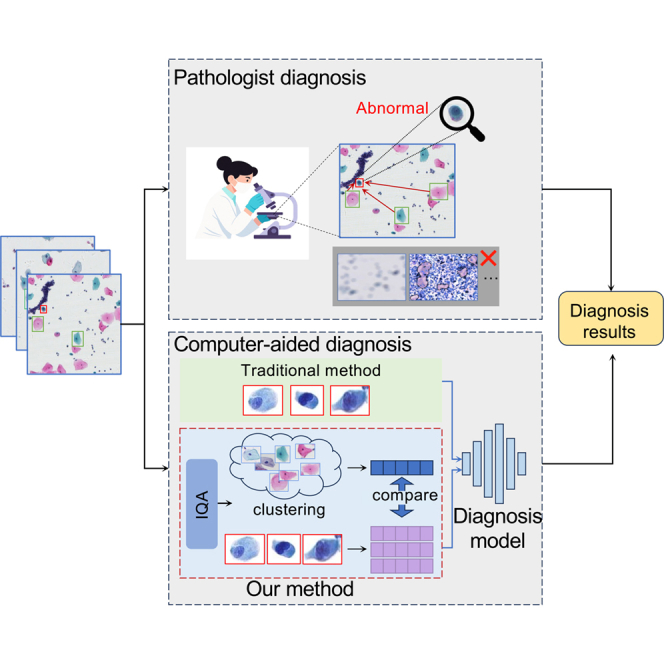

## Introduction

Cervical cancer remains a leading cause of female mortality globally, with approximately 661,000 new cases and 348,000 deaths reported in 2022.^1^^,^^2^ While early screening significantly reduces morbidity and mortality,^3^ traditional manual evaluation is labor-intensive, subject to inter-observer variability, and difficult to scale. Consequently, it is important to develop efficient and automated methods for cervical cancer diagnosis.^4^^,^^5^^,^^6^

With the rapid development of digital pathology and deep learning, many computer-aided diagnosis methods for cervical cancer have been proposed.^7^^,^^8^^,^^9^^,^^10^^,^^11^ Recent studies often perform whole slide image (WSI) classification by aggregating detected abnormal cells.^12^^,^^13^^,^^14^^,^^15^^,^^16^ However, these approaches rely heavily on accurate cell-level classification, while obtaining fine-grained pixel annotations remains challenging due to the massive size of WSIs. Since Ilse et al.^17^ introduced attention-based deep multiple instance learning (MIL) combined with convolutional neural networks in 2018, weakly supervised MIL has become the mainstream approach for WSI classification.^18^^,^^19^^,^^20^^,^^21^^,^^22^ Representative studies include the two-stage MIL strategy of Xie et al.,^23^ hierarchical MIL with class attention proposed by Jin et al.,^24^ and cross-scale MIL introduced by Deng et al.^25^ Although these methods achieve promising performance,^26^^,^^27^ several limitations still remain.

First, image quality problems may result in analytical failures or higher false-positive rates.^28^^,^^29^^,^^30^^,^^31^ In clinical practice, WSIs often show quality problems such as artifacts, blur, and uneven staining caused by variations in acquisition devices and environmental interference.^30^^,^^32^^,^^33^ However, most existing methods assume high-quality inputs and neglect the adverse impact of low-quality images on diagnostic performance. Second, the criteria for identifying abnormal cells vary across individuals due to physiological and personal differences. A cell type considered abnormal in one patient may appear normal in another. Nevertheless, many methods focus solely on abnormal cell detection without modeling such inter-patient variability, which can result in misclassification and reduced diagnostic reliability.

**Figure 1:**
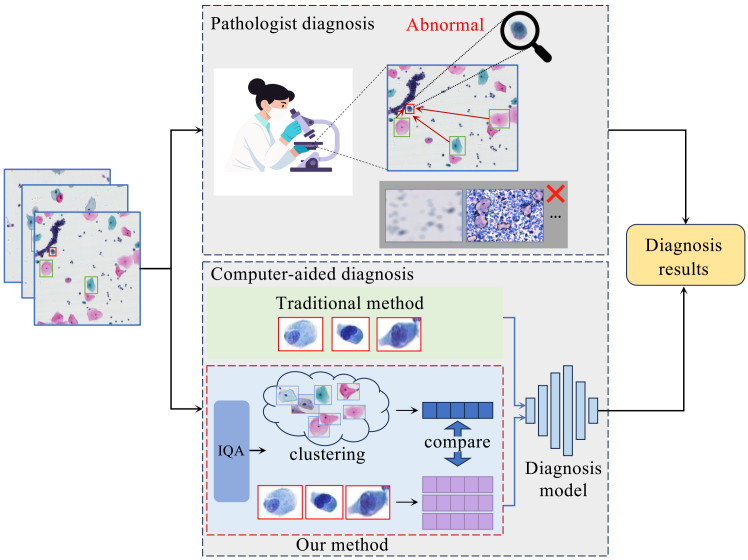
Schematic illustration of cervical cell comparative learning The upper part shows the pathologist’s diagnostic methods, and the lower part shows a comparison between the general method and our method.

To address these limitations, we propose a WSI classification method that integrates image quality assessment (IQA) into classification and imitates the diagnostic method of pathologists, as shown in Figure 1. According to TBS diagnostic guidelines, pathologists first evaluate sample quality and only diagnose satisfactory samples. Moreover, diagnosis is often based on comparing suspected abnormal cells with normal cells. Therefore, we incorporate IQA to guide the model toward high-quality regions while suppressing low-quality ones. To emulate this comparative strategy, by extracting normal cell prototypes from the same WSI as an internal reference, the model contrasts suspected abnormalities against a patient-specific baseline. This comparison, enforced by an instance contrastive loss, effectively differentiates true abnormalities from physiological variations.

## Results

### Demographic characteristics

**Figure 2:**
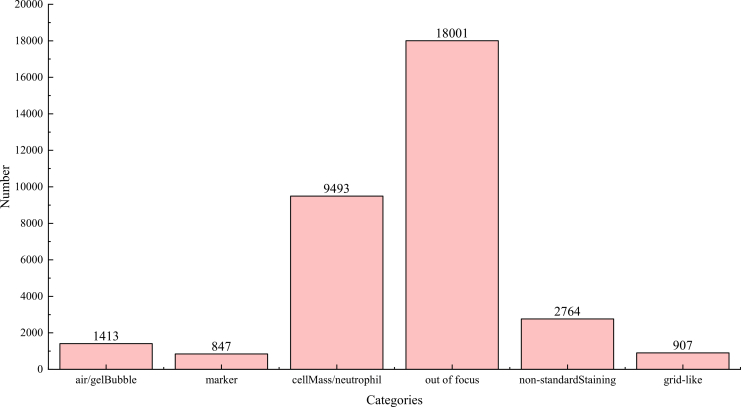
Distribution of the image quality assessment dataset The chart quantifies samples across these artifact categories, with out of focus (18,001) and cell mass/neutrophil (9,493) being the most prevalent classes.

This multicenter study enrolled 2,434 female patients with 2,434 WSIs from five hospitals in Harbin, including 1,518 normal WSIs and 916 abnormal WSIs. Data from 1,904 patients (1,904 WSIs) across the five hospitals were used for model development, while the remaining 530 patients (530 WSIs) from the same centers constituted the external validation set. The patient age ranged from 20 to 75 years, with a peak age of 30–55 years (74.2%, 680 WSIs). This finding aligns with the documented high incidence of cervical cancer. The distribution of the dataset information is detailed in Tables 1 and 2 and Figure 2.

**Table 1:** Distribution of the dataset

Medical centers	Normal	Abnormal	Total
The first affiliated hospital of Heilongjiang University of Chinese Medicine	25	53	78
The fourth affiliated hospital of Harbin Medical University	470	597	1067
The sixth affiliated hospital of Harbin Medical University	120	44	164
Cancer Hospital affiliated to Heilongjiang Medical University	126	62	188
Harbin Jiarun Hospital	777	160	937
WSIs with quality problems	68	189	257
Total	1518	916	2434

The experimental dataset consists of a total of 2,434 samples, categorized into 1,518 normal and 916 abnormal cases, respectively.

**Table 2:** Details of the cell detection dataset

-	Train		Test	
	Patch	Cell	Patch	Cell
Normal	1,204	17,842	305	4,579
Abnormal	55,959	128,185	13,986	32,586
Total	57,163	146,027	14,291	37,165

The table presents the statistical distribution of the dataset, specifying the number of patches and cell images included in the training and test sets, respectively.

### Model performance

We evaluated the effectiveness of the proposed method using clinical data as the training and validation dataset, based on real-world medical requirements. Our method outperforms current mainstream state-of-the-art methods across several evaluation metrics, including RNN-MIL,^34^ Snuffy,^35^ DGMIL,^36^ ABMIL,^17^ attFPN,^14^ Dual-path,^15^ DSMIL,^5^ DTMIL,^37^ TransMIL,^38^ PHIM-MIL,^23^ and SeLa-MIL^39^ under the same settings, which fully verifies the effectiveness and advancement of the proposed method. In particular, our method achieves the highest performance in the metrics of accuracy and area under the receiver operating characteristic curve (AUC). Specifically, our method achieves an accuracy of 90.23%, which is 1.93% higher than the classical TransMIL (88.30%). Notably, our method reaches 93.66% in the AUC metric, which is significantly higher than that of other compared methods, such as Dual-path (90.66%), TransMIL (92.66%), and ABMIL (89.52%), indicating that the proposed method is more stable and robust in terms of overall classification performance (see Table 3).

**Table 3:** Performance metrics of different WSI classification models

Model	Accuracy	Sensitivity	Specificity	AUC
Cheng et al.^34^	0.8750	0.7674	0.7980	0.8696
Jafarinia et al.^35^	0.8264	0.7937	0.8053	0.8762
Qu et al.^36^	0.6530	0.5200	0.6433	0.6134
Ilse et al.^17^	0.8642	0.8202	0.8639	0.8952
Cao et al.^14^	0.8361	0.7444	0.8763	0.8361
Lin et al.^15^	0.8434	0.6935	0.9095	0.9066
Li et al.^5^	0.8509	0.8104	0.8419	0.8860
Zhang et al.^37^	0.8453	0.8256	0.8237	0.8956
Shao et al.^38^	0.8830	0.8610	0.8702	0.9266
Xie et al.^23^	0.8321	0.7934	0.8154	0.8689
Ma et al.^39^	0.8571	0.8711	0.7677	0.8333
Our method	0.9023	0.8292	0.8512	0.9366

Different models are assessed using four key quantitative metrics: Accuracy, sensitivity, specificity, and AUC.

To prove the statistical significance of our method, improvements in accuracy and AUC values were assessed using 95% confidence intervals estimated by the DeLong method. Compared with TransMIL, our method achieved a 1.93% improvement in accuracy, which was statistically significant, and also yielded a higher AUC with non-overlapping confidence intervals (see Table 4).

**Table 4:** Pairwise comparison of our method against baseline models using DeLong confidence intervals, 95% CI

Method	Accuracy	AUC	ΔAUC vs. Ours
Ilse et al.^17^	0.8642[0.8430, 0.8854]	0.8952(0.8821–0.9243)	+0.0325
Shao et al.^38^	0.8830[0.8631, 0.9029]	0.9266(0.9077–0.9455)	+0.0011
Our method	0.9023[0.8839, 0.9207]	0.9366(0.9242–0.9538)	-

### Ablation performance

**Figure 3:**
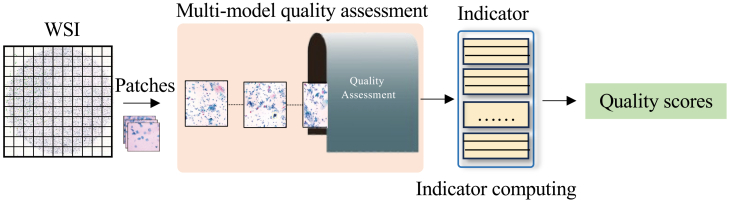
Overview of the quality assessment model The proposed framework consists of four stages: (1) The input WSI is tessellated into non-overlapping patches; (2) these patches are fed into the multi-model quality assessment module to extract features; (3) a stack of metrics is generated from the assessment; and (4) the final quality scores are obtained through indicator computing.

**Figure 4:**
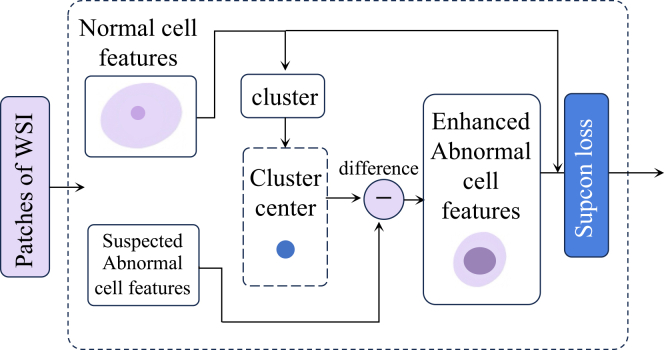
Normal/abnormal cell comparison enhancement module The module enhances suspected abnormal cell features by computing their difference from a normal cell cluster center, optimized via Supcon loss.

**Figure 5:**
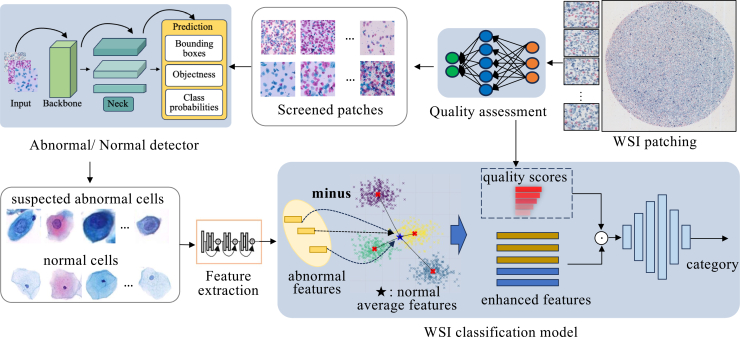
Pipeline of the proposed method Following quality assessment, image patches with extremely low quality were excluded in the initial screening. Subsequently, a normal/abnormal cell detection model was applied to identify suspected cells, and finally, the features of these detected cells were fed into our contrastive learning-based cell classification model.

To evaluate the effectiveness of each component in our proposed framework, we conducted a series of ablation studies on the benchmark cervical cytopathology WSI dataset. The overall results are reported in Table 5, where QISM indicates the quality initial screening module (see Figure 3), CFCM indicates the cell feature comparison module (see Figure 4), and QAM indicates the quality-aware module in MIL with Supcon Loss (model framework is shown in Figure 5).

**Table 5:** The ablation experimental results of each component of our method, where QISM indicates the quality initial screening module, CFCM indicates the cell feature comparison module, and QAM indicates the quality-aware module in multiple instance learning with Supcon loss

Baseline	QISM	CFCM	QAM	Accuracy	Sensitivity	Specificity	AUC
Attention-based MIL	–	–	–	0.7962	0.7072	0.8204	0.8749
	✓	–	–	0.8566	0.8206	0.8596	0.9265
	–	✓	–	0.8679	0.8513	0.8493	0.9324
	–	–	✓	0.8736	0.8317	0.8752	0.9384
	✓	✓	–	0.8855	0.8361	0.8750	0.9366
	✓	✓	✓	0.9023	0.8292	0.8512	0.9366

The results show that each component improves the model performance. The baseline model, attention-based MIL equipped with the QISM, achieves higher precision, indicating the effectiveness of the quality initial screening. CFCM and QAM further improve the classification performance, demonstrating the effectiveness of cell feature comparison and quality assessment, respectively. Finally, our method gets an accuracy of 0.9023, a sensitivity of 0.8292, a specificity of 0.8512, and an AUC of 0.9366 (see Table 5).

We use the *K*-means clustering algorithm to obtain the general features of normal cervical cells, where each cluster contains similar normal cells. Different values of *K* can have varying effects on the model. In this paper, we conduct experiments on our dataset to explore the effect of the number of *K* on model performance.

**Figure 6:**
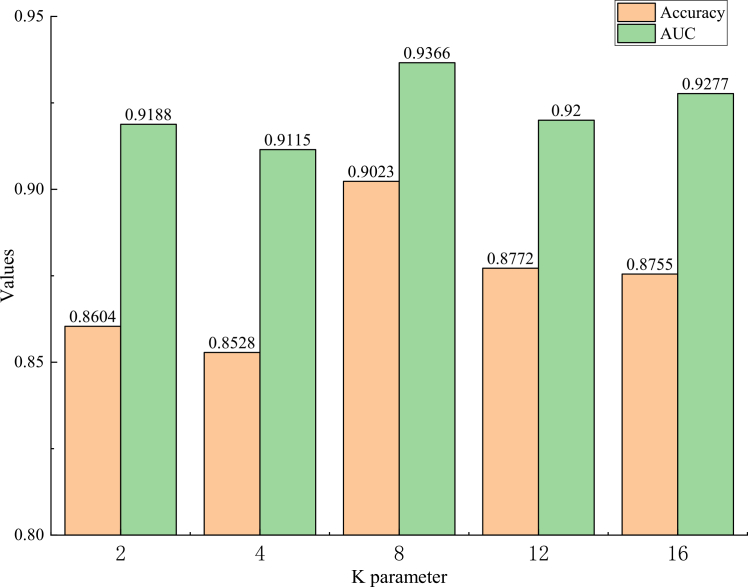
Impact of the hyperparameter K on model performance The chart compares accuracy and AUC scores across varying *K* values (2,4,8,12,16). The model achieves optimal performance at *K* = 8, yielding the highest accuracy of 0.9023 and AUC of 0.9366.

As shown in Figure 6, our model performs best when the *K* parameter is 8. We assert that when *K* is too large, each cluster represents only a small portion of normal cells. Conversely, if *K* is too small, the clusters may not sufficiently capture the diversity and complexity of normal cells. This results in a model that is less effective at identifying abnormal cells through comparison with normal cells. Therefore, K = 8 is a reasonable choice between performance and efficiency because of its moderate computational overhead, while maintaining high performance.

### Visualizations of model predictions and error analysis

**Figure 7:**
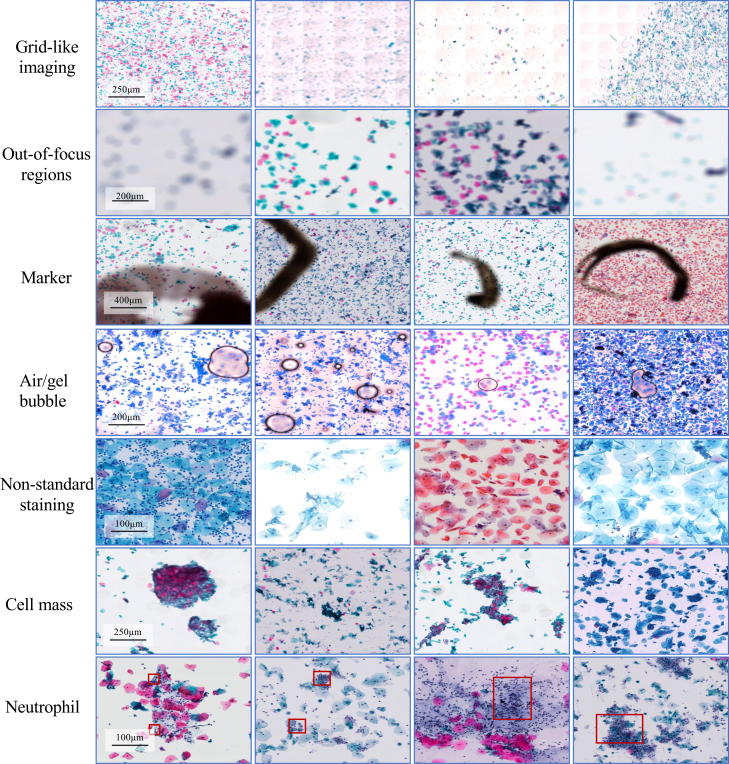
Examples of quality problems in cervical cytopathology images To better represent samples of quality problems, we use a different scale bar.

**Figure 8:**
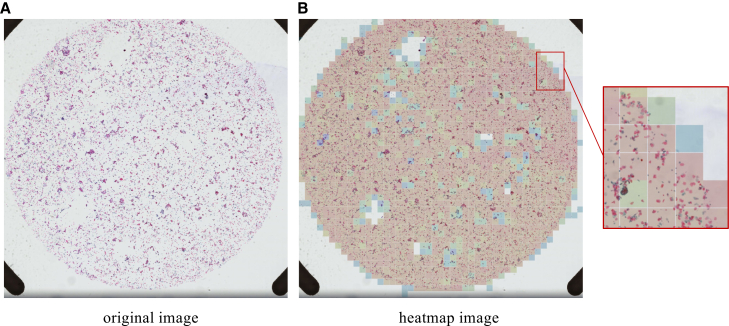
Heatmap of WSI quality scores Red indicates higher quality, blue indicates lower quality.

We introduce an IQA module based on the Bethesda System (TBS) standard and image content analysis. It initially filters out low-quality regions that have a significant negative impact on classification, while incorporating quality scores into multi-instance learning so that the model pays more attention to image regions with diagnostic value, significantly reducing the interference of artifacts, blurring, and other factors in classification accuracy (see Figure 7). Figure 8 visualizes the quality scores ranging from 0 to 1.

**Figure 9:**
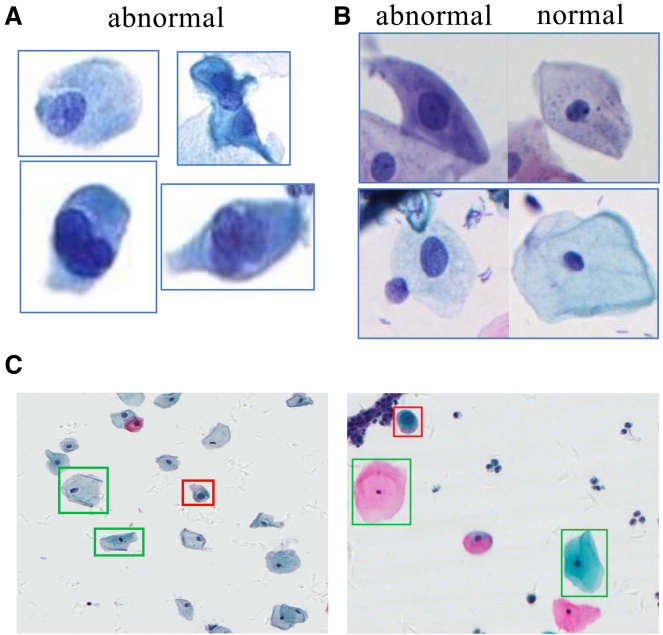
Abnormal cells for cervical WSI (A) Abnormal cells in different WSIs. (B) Abnormal and normal cell in the same WSI. (C) The red box indicates the abnormal cell, and the cells in the green boxes are surrounding reference cells.

**Figure 10:**
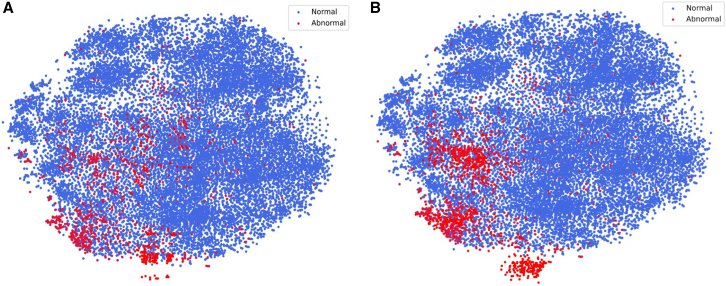
T-SNE visualization (A) The original feature distribution. (B) After cell comparison. After using our method, similar instances are more tightly clustered, and the separation between dissimilar instances is more pronounced.

**Figure 11:**
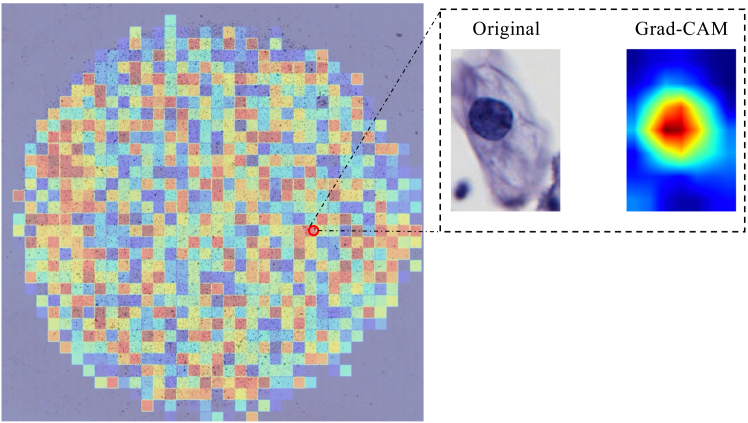
Visualization of key areas of interest for slide-level predictions The figure displays the original image alongside its corresponding Grad-CAM activation map, where red regions denote high importance, and blue regions represent ignored background information.

By constructing feature contrasts between normal and suspected abnormal cells within the same WSI (see Figure 9), we enhance the recognition of abnormal cells in the feature space, thus effectively addressing the challenge of cervical cells that are highly similar in their morphologic manifestations, as shown in Figure 10, which demonstrates the feature distribution. To improve interpretability, we visually present slide-level predicted areas on representative patches. As shown in Figure 11, the model primarily focuses on abnormal cell populations and diagnostically relevant regions that are consistent with the areas of interest identified by experienced cytopathologists. This finding suggests that our method not only provides accurate and interpretable predictions.

### Reader performance

To further evaluate the proposed method, we recruited five experienced cytopathologists for sample screening. Three cytopathologists (C1–C3) have over 5 years of experience, while the other two (C4–C5) have over 3 years (see Table 6). Among the annotators, three were senior pathology technicians, and two were associate chief physicians. The inter-rater agreement among experts, as measured by Fleiss' Kappa, reached 0.87. To ensure the transparency and reproducibility of the annotation process, a standardized procedure was followed: each annotator first performed the labeling independently, followed by cross-checking. For cases with disagreements, the annotators reached a final consensus through discussion and by consulting reference materials.

**Table 6:** Comparative results of the proposed method and cytopathologists

Model	Accuracy	Sensitivity	Specificity
Cytopathologist 1	0.8043	0.7046	0.8623
Cytopathologist 2	0.7850	0.7200	0.8200
Cytopathologist 3	0.7500	0.7050	0.7902
Cytopathologist 4	0.8000	0.7403	0.8156
Cytopathologist 5	0.7351	0.7005	0.7752
Cytopathologist mean	0.7749	0.7141	0.8127
Our method	0.9023	0.8292	0.8512

The performance is evaluated based on accuracy, sensitivity, and specificity. The table lists results for five individual experts, their average (cytopathologist mean), and our proposed model.

**Figure 12:**
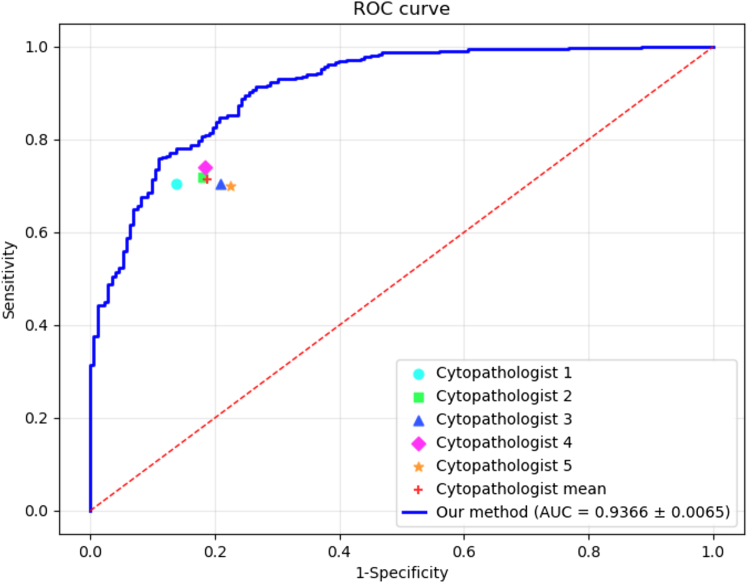
Performance comparison between the proposed method and cytopathologists for cervical WSI classification The blue curve represents our method. Discrete markers denote the performance of five cytopathologists and their mean value (red cross), illustrating that the model outperforms the average human expert.

The average accuracy of all pathologists is 0.7749, the average sensitivity is 0.7141, and the average specificity is 0.8127. Compared to the pathologists, the proposed method improved accuracy, sensitivity, and specificity by 0.1274, 0.1151, and 0.0385, respectively. The significant performance improvement not only shows the effectiveness of the method but also shows the great potential of deep learning technology in cervical cytopathology WSI classification. Our proposed method achieves higher ROC performance than each of the 5 cytopathologists individually across a range of operating thresholds (see Figure 12). The ROC curves demonstrate that our model consistently exhibits better sensitivity-specificity trade-offs compared to experts with varying experience levels.

## Discussion

In this study, we proposed a pathologist-inspired framework for cervical WSI classification that significantly improves diagnostic robustness. By integrating IQA with a patient-specific cell comparison strategy, our model effectively addresses the critical real-world challenges of variable image quality and inter-individual cellular heterogeneity.

The key innovation of our work is its emulation of a pathologist’s diagnostic process. Our framework is the first in this field to use an IQA module to weight instances within a MIL architecture, enabling the model to prioritize high-fidelity regions and mitigate artifacts. This quality-aware approach is crucial for reliable performance across different clinical settings. Furthermore, by establishing a “normal” cellular baseline within each WSI for comparison, our model accurately identifies abnormal cells while accommodating patient-specific morphological variations. This comparative strategy, enhanced by supervised contrastive learning, sharpens the distinction between normal and abnormal cells. Experiments on 2,434 WSIs from five medical institutions demonstrate that our method achieves superior performance in real-world scenarios, significantly outperforming state-of-the-art methods by 1.93% in average overall accuracy.

### Limitations of the study

Despite its strong performance, our study has several limitations. First, the IQA module may not be robust against rare or novel artifacts not encountered during training. Future work could focus on more adaptive quality control mechanisms. Second, our cell comparison strategy depends on the presence of sufficient normal cells to establish a reliable baseline, which may be a constraint in slides with extensive lesions. Third, this was a retrospective study. A prospective clinical trial is necessary to validate the model’s real-world utility, cost-effectiveness, and seamless integration into clinical workflows. Finally, like many deep learning systems, our model’s decision-making process is not fully transparent. Incorporating more advanced explainable AI techniques could enhance clinician trust and adoption.

## Resource availability

### Lead contact

Requests for further information and resources should be directed to and will be fulfilled by the lead contact, Jian Qin (qinjian@ahut.edu.cn).

### Materials availability

This study did not generate new unique reagents.

### Data and code availability

• All data reported in this paper will be shared by the lead contact upon request.
• All original code has been deposited on GitHub and is publicly available as of the date of publication. The repository URL is listed in the key resources table.
• Any additional information required to reanalyze the data reported in this paper is available from the lead contact upon request.

## Acknowledgments

This work was supported by the 10.13039/501100001809National Natural Science Foundation of China under grant 62473111 and the Science and Technology Innovation Tackle Plan Project of Maanshan under grant 2024RGZN001.

## Author contributions

Conceptualization, L.K. and J.Q.; methodology, L.K.; investigation, L.K., J.W., and Y.H; writing – original draft, L.K.; writing – review and editing, L.K., J.W., and Y.H.; funding acquisition, Y.H. and J.Q.; resources, Y.H.; supervision, J.W., Y.H., and J.Q.

## Declaration of interests

The authors declare no competing interests.

## STAR★Methods

### Key resources table

**Table:** 

REAGENT or RESOURCE	SOURCE	IDENTIFIER
**Software and algorithms**		

Python version 3.10	Python Software Foundation	https://www.python.org
Pytorch version 2.0.1	PyTorch Foundation	https://pytorch.org
SimCLR	SimCLR website	https://doi.org/10.5555/3524938.3525087
yolov5	Yolov5 website	https://doi.org/10.1109/CVPR.2016.91

**Deposited data**		

Our code	This study	https://github.com/kqsgdka/cell-comparative-MIL-guided-by-IQA-for-cervical-WSI

**Other**		

NVIDIA GeForce RTX 3090	Nvidia Corp., Santa Clara, California, USA	N/A

### Experimental model and study participant details

#### Ethics statements

This study was approved by the Institutional Review Board of Harbin Institute of Technology (No. HIT-2024068). Written informed consent was obtained from participants using the IRB-approved consent form.

### Method details

#### Datasets collection

This study collected patient samples of female patients aged 20 to 75 years who underwent cervical cancer screening at five hospitals in Harbin city between December 2024 and June 2025. Inclusion criteria for the images were: 1) age ≥20 years old; 2) vailability of a definitive diagnostic report (ranging from normal/NILM to precancerous lesions and cervical cancer); 3) single sample per individual to ensure independent observations.; 4) A standard WSI sample or a WSI sample with some quality issues. Exclusion criteria for the images were: 1) age <20 years old; 2) histopathological images (to maintain focus on cytological screening); 3) edundant samples from the same patient; 4) samples with insufficient cellularity for diagnostic evaluation (e.g., squamous cells). The development of the model incorporated a total of 2,434 WSIs from 2,434 patients from five hospitals, including 257 WSIs with quality problems (e.g., artifacts, blur, and uneven staining). Data from 1,904 patients (1,904 WSIs) across five hospitals were used for model development, while the remaining 530 patients (530 WSIs) from the same five centers constituted the external validation set. The distribution of the dataset information is detailed in Table 1.

#### Datasets description

We evaluate our method on a dataset of 2,434 WSIs collected from 5 medical centers, including 257 with quality problems, as detailed in Table 1. The slides were scanned by using KonFoong Biotech International (KF-PRO-020) with a resolution of 0.25 *μ* m/pixel at ×40 magnification, HuNan Gokvision Intelligent Technology (ZT-S240) at 0.22 *μ* m/pixel at ×20 magnification, and WISLEAP (WS-10) at 0.23 *μ* m/pixel at ×40 magnification. In addition to variations in scanner types and imaging settings, the dataset also exhibits natural differences in staining protocols across centers, further reflecting real-world diversity. This multi-center, multi-scanner, and multi-staining dataset ensures robustness and high relevance to clinical applications. The study was conducted in compliance with the Declaration of Helsinki and received approval from the Harbin Institute of Technology Institutional Review Board (Approval No. HIT-2024068; Date of Approval: 2024-09-26).

To train the quality assessment model, we collect several image patches with various resolutions of 256 × 256, 512 × 512, and 1024 × 1024. The training set for metrics calculation contains 33,425 patches. The specific distribution of the dataset in Figure 2.

To train the Yolov5 for normal and abnormal cells, we annotate normal and abnormal cells in image patches with a resolution of 1024 × 1024. These cell-level image patches are derived from different cervical cytopathology WSIs. The annotation process was performed by three annotators with backgrounds in cytopathology, who received standardized training for this task. To ensure annotation quality, we implemented a consensus protocol where initial annotations were made independently, and any discrepancies were adjudicated by a senior pathologist to establish the final ground truth. The inter-observer agreement, measured on a randomly selected subset of 1,000 cell annotations, yielded a Fleiss' Kappa value of 0.87, indicating good consensus among the annotators. The specific distribution of the dataset is shown in Table 2. The training set for abnormal cell detection contains 55,959 patches, comprising a total of 128,185 abnormal cell annotations. In contrast, the training data for normal cell detection includes 1,204 patches, with a total of 17,842 normal cell annotations.

#### An architerture of the method

The workflow of our proposed method is illustrated in Figure 5. First, WSIs are divided into non-overlapping patches of size 1024 × 1024 pixels and screened by a pretrained quality assessment model to filter out low-quality regions. Second, two independently pretrained YOLOv5 models are used to detect abnormal cells and normal cells within each patch. Third, in the Normal-Abnormal Cell Comparison Module, these features are refined using Supervised Contrastive (SupCon) loss. This step explicitly distances abnormal features from the computed normal prototypes in the embedding space. Finally, the enhanced features are aggregated via an attention-based MIL network, where the pre-computed quality scores serve as instance weights to generate the final slide-level diagnosis.

##### Image quality control

We propose a quality assessment model for cervical cytopathology WSIs based on TBS diagnostic standards^40^ and image content analysis, as illustrated in Figure 3. This model is trained in a supervised manner using a large number of WSIs with annotated quality scores. The scoring criteria encompass multiple quality dimensions, including artifacts, blur, and uneven staining, as illustrated in Figure 7. To ensure diagnostic reliability, a conservative quality threshold is set in the method according to the experience of pathologists. Only patches with quality scores exceeding this threshold are selected for subsequent processing, thereby minimizing the impact of low-quality regions on the final classification performance.

The evaluation pipeline begins with cell localization using a pretrained object detection model, followed by cell counting. Next, a clarity assessment module evaluates the focus quality of each patch. A segmentation model is then applied to identify and delineate individual cells, cell clusters, and potential interfering artifacts such as markers, air/gel bubbles. Subsequently, an adaptive stain separation technique is applied to evaluate the consistency and standardization of staining. Finally, quality scores from all dimensions are integrated to compute a comprehensive patch-level quality score. The performance on an expert-labeled dataset has been validated with an AUC of 0.93 and Cohen’s *κ* of 0.82. Full implementation details and validation results are provided in our recent work.^41^

##### Cell detection and feature extraction

In cervical WSIs, which are composed of hundreds of thousands of cells, the number of abnormal cells is very small, often numbering only a few to a few dozen per slide. These abnormal cells exhibit a sparse and discrete spatial distribution, leading to a severe class imbalance. This imbalance makes it challenging for traditional WSI analysis strategies to effectively localize key diagnostic information. Therefore, accurate detection of potential abnormal cells before WSI classification becomes a critical step to improve the overall diagnostic performance.

In our proposed method, we use YOLOV5^42^ to detect abnormal cells in the image patch following initial quality-based screening. Compared to two-stage detectors such as Faster R-CNN and Mask R-CNN, the one-stage YOLOv5 model provides a superior balance between speed and accuracy. YOLOv5 adopts CSPDarknet53 as its backbone network, incorporates PANet for multi-scale feature fusion, and employs AutoAnchor generation and mixed precision training, thereby reducing GPU memory consumption while maintaining detection performance and enhancing deployment efficiency. Considering the extreme rarity of abnormal cells in cervical cytopathology images, with abnormal/normal sample ratios reaching as high as 1:10,000, we independently constructed and annotated a dedicated abnormal cell detection dataset to train a YOLOv5 model specifically for identifying abnormal cells.

To extract discriminative cell features effectively, we employ SimCLR,^43^ a state-of-the-art self-supervised learning approach, as the pretraining framework for our cell encoder. SimCLR learns semantic representations by maximizing the similarity between different augmented views of the same image, without the need for manual annotations. During training, two augmented views are generated for each cell image through a series of stochastic transformations and encoded using a shared neural network. A contrastive loss function is then applied to optimize the embeddings. We use a large number of non-overlapping image patches cropped from cervical WSIs as training data, enabling the model to learn generalizable and robust representations of cells. These embeddings serve as high-quality inputs for downstream tasks.

##### Cell comparison enhance

From a clinical perspective, a cell type considered abnormal in one patient may appear normal in another, as shown in Figures 9A and 9B. Thus, pathologists often rely on morphological comparisons with normal cells to assist in the identification of abnormal ones, as shown in Figure 9C. Inspired by this diagnostic strategy, we design a Normal-Abnormal Cell Comparative Enhancement Module, which incorporates representative normal cells from the same WSI as references to effectively highlight the distinguishing features of abnormal cells. As illustrated in Figure 4, this module uses normal cells a as the reference, which further enhances the discrimination of abnormal cells in the feature space.

We first extract normal and suspected abnormal cells from cervical cytopathology WSIs and perform pretraining on a large-scale unlabeled dataset to learn generalized feature representations of cells. In practical applications, using the same normal cell as a reference for all WSI is unreliable, while referencing all normal cells is both unnecessary and computationally inefficient. Moreover, due to due to inter-individual differences in cervical cell morphology, many normal cells may lack representative characteristics. In clinical diagnosis, pathologists typically select one or two prototypical normal cells as references. Following this principle, we perform clustering analysis on the normal cell features within each WSI to obtain cluster centers, and compute the average feature representation of normal cells as the reference. We then calculate the feature differences between suspected abnormal cells and the averaged normal cell representation, thereby highlighting their discriminative characteristics.

To further enhance abnormal cell recognition, we introduce an instance-level supervised contrastive loss to optimize the feature space between normal and abnormal cells. Specifically, contrastive pairs are constructed based on cell-level annotations: instances from the same class (e.g., abnormal-abnormal or normal-normal) are treated as positive pairs, while instances from different classes are treated as negative pairs. During training, embeddings of abnormal cells are pulled closer together, while the distance between abnormal and normal cells is explicitly increased. This strategy enforces intra-class compactness and inter-class separability, effectively alleviates the problem of abnormal cells being diluted in the feature space, and reduces confusion between categories in the embedding representation, thereby improving the overall classification accuracy and robustness of the model. Supcon Loss is calculated as follows:

(Equation 1) $$L_{supcon}=\sum_{i\in I}\frac{1}{\left|P\left(i\right)\right|}\sum_{p\in P\left(i\right)}-\log\frac{\exp\left(z_{i}\cdot z_{p}/\tau \right)}{\sum_{a\in A\left(i\right)}\exp\left(z_{i}\cdot z_{a}/\tau \right)}$$

where *I* is the indexed collection of all instances, *P*(*i*) is a collection of positive instances in the same category as instance *i*, *A*(*i*) contains all instances except *I* itself (positive + negative), *z*_*i*_ and *z*_*p*_ are embedding vectors for instance *i* and positive instance p respectively, *τ* is the temperature coefficient that controls the smoothness of the distribution.

##### Quality-aware attention-based MIL

To effectively integrate instance-level feature information from cervical cytopathology images, we construct an attention-based MIL classification framework. The framework is suitable for weak supervision scenarios in pathological images, such as insufficient label granularity and missing annotation. By introducing the image quality-aware mechanism, the discriminative ability and robustness of the model are significantly improved.

Specifically, we utilize patch-level quality scores as instance weights to dynamically modulate their contributions to the final classification decision. This design guides the model to focus on high-quality regions while suppressing the influence of low-quality information, thereby reducing prediction noise. In the overall pipeline, WSI undergoes the steps, including quality assessment, cell detection, feature extraction, and cell comparative enhancement, and finally generates patch-level cell feature vectors. These features are then fed into a quality-aware attention-based MIL module for feature aggregation and final WSI-level classification.

In the MIL framework, a set of training samples is called a bag *X* = *x*_1_, …,*x*_*k*_. It contains multiple unordered instances. In our proposed model, each WSI is considered as a bag *X* = *x*_1_, …,*x*_*k*_. Where *x*_*i*_ denotes the *i*-th cell feature, *K* denotes the number of instances, and *K* can be different for each WSI. Different from the traditional MIL, this paper introduces the set of quality scores: *S* = *s*_1_, …,*s*_*k*_ as a weighting factor to regulate the influence degree of each instance feature. First, the original features are mass-weighted to obtain a new bag feature representation *X*′:

(Equation 2) $$x_{i}^{\prime }=x_{i}s_{i}$$

(Equation 3) $$X^{\prime }=x_{1}^{\prime },...,x_{k}^{\prime }$$

where *X*′ is the WSI bag feature that fuses the image quality information, $x_{i}^{\prime }$ prime is the *i*-th instance feature that fuses the image quality score, *i*∈[0,*k*], *k* is the number of instances.

Next, we use a pooling strategy based on the attention mechanism to weight and aggregate the instance features to form a bag-level representation vector:

(Equation 4) $$z=\sum_{k=1}^{K}a_{k}x_{k}$$

The attention weight *a*_*k*_ is computed as follows:

(Equation 5) $$a_{k}=softmax\left(W^{T}\;\max\left(LineNorm\left(Vx_{k}^{T}\right),0\right)\right)$$

where *a*_*k*_ denotes the attention weigh of the *k*-th instance, $W\in R^{L\times 1}$ and $V\in R^{L\times M}$ are the learnable parameters of the linear layer. *LineNorm*(·) denotes the layer normalization operation, *max*(·,0) is ReLU activation function. This mechanism effectively suppresses interference from redundant or unreliable instances, allowing the model to focus more on diagnostically relevant cellular regions. Eventually, the bag representation vector *z* will be input to the fully connected layer with the softmax classifier to obtain the classification prediction probability *p*∈*R*^*C*^, where *C* denotes the number of categories.

#### Overall loss function

To further improve the discriminative power of the model, we jointly optimize the MIL classification loss with the instance-level SupCon Loss. Different weights are used to control the constraints and contributions of instance-level loss and package-level loss during training. The overall loss function of our cervical cell WSI classification model during training is defined as follows:

(Equation 6) $$L=\lambda L_{bag}+\left(1-\lambda \right)L_{supcon}$$

where *λ* is the regulation weight, which is used to balance the effects of the two types of losses. *L*_*bag*_ denotes the regular cross-entropy loss, defined as:

(Equation 7) $$L_{bag}=-\sum_{i=1}^{C}y_{i}\;\log\;p_{i}$$

where *p*_*i*_ and *y*_*i*_ are the predicted result and ground truth, *C* is the number of categories. *L*_*supcon*_ improves the model’s ability to sense subtle morphological differences by optimizing the feature-embedding relationship between abnormal and normal cells, as defined in the previous section.

### Quantification and statistical analysis

We implement the model using the PyTorch framework. All experiments and statistical analysis of data in this study were performed using Python 3.10, PyTorch 2.0.1, and CUDA 11.8 on an NVIDIA GeForce RTX 3090. To ensure reproducibility, we fixed the random seed to 42. We preprocess the dataset by cropping each WSI into 512 × 512 non-overlapping image patches at 20× magnification, forming a bag. All statistical details, including estimates of performance metrics, visualization, and 95% confidence intervals, are reported in the results section, and corre-sponding table legends.

Diagnostic performance was evaluated at the image level using classification performance metrics. The training set analyzed in this study are summarized in Tables 1 and 2. Classification performance was assessed using accuracy, sensitivity, specificity, and area under the receiver operating characteristic curve (AUC). These performance metrics are reported as point estimates paired with 95% confidence intervals in the results section and corresponding tables.

The statistical analysis incorporated rigorous methods to ensure robust inference. Bootstrap resampling with 1,000 iterations was used to calculate 95% confidence intervals(95% CI).
